# The application of internal traction technique in retroperitoneal robot-assisted partial nephrectomy for renal ventral tumors

**DOI:** 10.1186/s12957-022-02684-1

**Published:** 2022-06-23

**Authors:** Xiao-Lu Jiang, Kui OuYang, Rui Yang, Xiao-Yang Yu, Dian-Dong Yang, Ji-Tao Wu, Hong-Wei Zhao

**Affiliations:** 1 Department of Urology, The Affiliated Yantai Yuhuangding Hospital of Qingdao University, Yantai, Shandong China; 2 Affiliated Hospital of Weifang Medical University, School of Clinical Medicine, Weifang Medical University, Weifang, China; 3 Department of Urology, Affiliated Yantai Yuhuangding Hospital of Qingdao University, NO. 20 East Yuhuangding Road, Yantai, 264000 Shandong China

**Keywords:** Robotic surgery, Partial nephrectomy, Renal ventral tumor, Retroperitoneal

## Abstract

**Background:**

For patients with prior intra-abdominal surgery or multiple arteries, the retroperitoneal robot-assisted partial nephrectomy (rRAPN) is a better choice. The renal ventral tumor poses an additional challenge due to poor tumor exposure. This study is determined to assess the feasibility of an internal traction technique (ITT) in rRAPN for the management of renal ventral tumors.

**Methods:**

From November 2019 to March 2021, a total of 28 patients with renal ventral tumor underwent rRAPN. All patients had prior abdominal surgery or multiple arteries. The ITT group (20 patients), which improved the tumor exposure by traction of the kidney with suture, was compared with the traditional technique group (8 patients) in terms of warm ischemia time, estimated blood loss and postoperative hospital stay, retroperitoneal drainage, R.E.N.A.L. score, and serum creatinine. Differences were considered significant when *P* < 0.05.

**Results:**

All rRAPN surgeries were successful without conversion to radical nephrectomy or open partial nephrectomy. The warm ischemia time was lower in the ITT group (17.10 min vs. 24.63 min; *P* < 0.05). Estimated blood loss in the traditional technique group was 324.88 ± 79.42 mL, and in the ITT group, it was 117.45±35.25 mL (*P* < 0.05). No significant differences with regard to postoperative hospital stay, retroperitoneal drainage, R.E.N.A.L. score, and serum creatinine were observed between both groups. Surgical margins were negative and no intraoperative complications occurred in all the patients. After 10 months of follow-up, no recurrence or metastasis occurred in all cases.

**Conclusion:**

ITT is a feasible, safe, and valid procedure in rRAPN for renal ventral tumors. Application of ITT improved the exposure and reduces warm ischemic time in comparison with the conventional procedure.

**Supplementary Information:**

The online version contains supplementary material available at 10.1186/s12957-022-02684-1.

## Background

Partial nephrectomy has gradually become a standard surgical procedure for the treatment of renal malignancy [1–3]. The robot-assisted partial nephrectomy is widely used in the surgical treatment of renal tumors and most commonly performed through a transperitoneal approach [4, 5]. However, for patients with prior intra-abdominal surgery or multiple arteries, the retroperitoneal approach is a better choice [6–9]. In retroperitoneal robot-assisted partial nephrectomy (rRAPN), dealing with renal ventral tumor with poor tumor exposure would be at risk of longer warm ischemic time (WIT) and more blood loss. Patients with complex renal masses were therefore often converted to open partial nephrectomy or radical nephrectomy [10, 11]. We developed an internal traction technique (ITT) to improve exposure, which is of great value for more effective tumor resection and renal reconstruction. In this article, we present this technique and evaluate its feasibility and efficacy in a retrospective case-control comparative study.

## Methods

### Patients

From November 2019 to March 2021, a total of 28 consecutive patients with renal ventral tumor (≤7cm) on computed tomography or magnetic resonance imaging underwent rRAPN. Seventeen cases had prior abdominal surgery and 11 cases had multiple renal arteries. No lymph nodes or renal vessels were involved in all tumors. Tumor complexity was evaluated according to the R.E.N.A.L. score [12–14]. Finally, ITT was performed in 20 cases with 12 cases that had prior abdominal surgery and 8 cases that had multiple renal arteries (ITT group). Eight cases underwent conventional rRAPN (traditional technique group). The metastatic cases were excluded by computed tomography, radionuclide bone imaging, or other specific scans according to clinical indication. Blood sample analysis was performed preoperatively and postoperatively. All procedures were performed by the same surgeon. The data of patients’ demographic characteristics, tumor sizes, R.E.N.A.L scores, preoperative laboratory results, warm ischemic time, estimated blood loss, operation-related complications, pathologic results, postoperative hospital stay, and retroperitoneal drainage were collected retrospectively. All the patients were followed postoperatively according to the recommendation of the EAU guideline [15].

The study was approved by Yantai Yuhuangding Hospital Ethics Committee. Written informed consent was obtained by the participants. The patients were all informed that their clinical data might be used in future study without invasion of privacy during hospitalization.

### Surgical technique

**Fig. 1:**
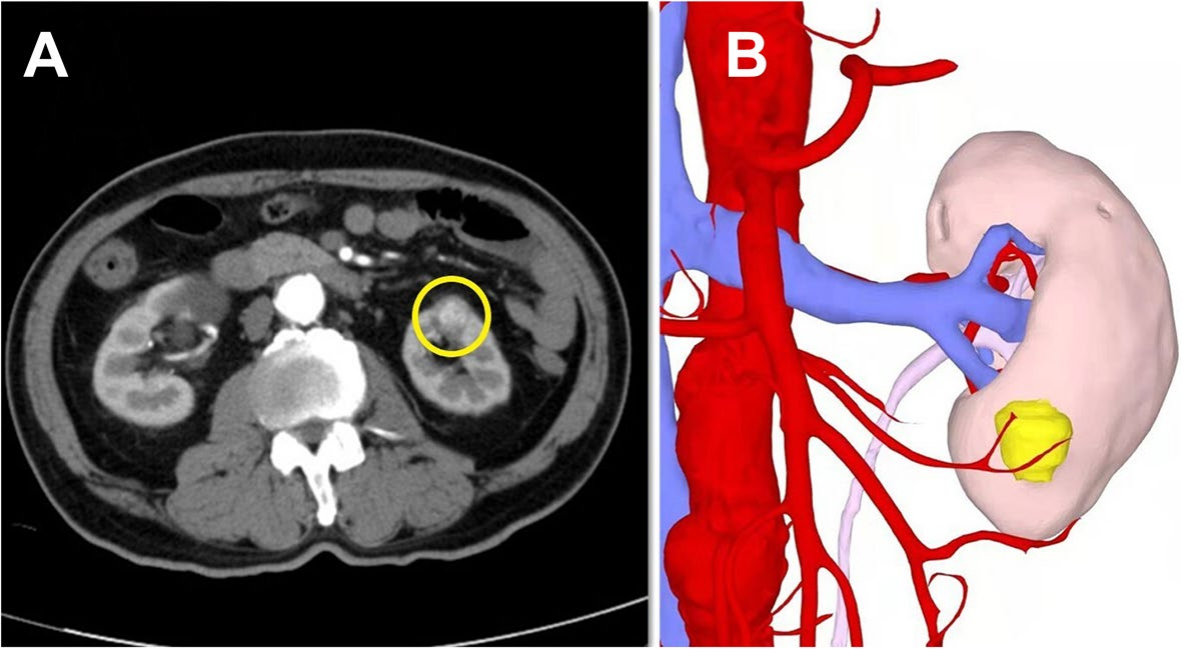
Ventral renal tumor (in yellow). **a** Computed tomography transverse view. **b** Three-dimensional reconstruction

**Fig. 2:**
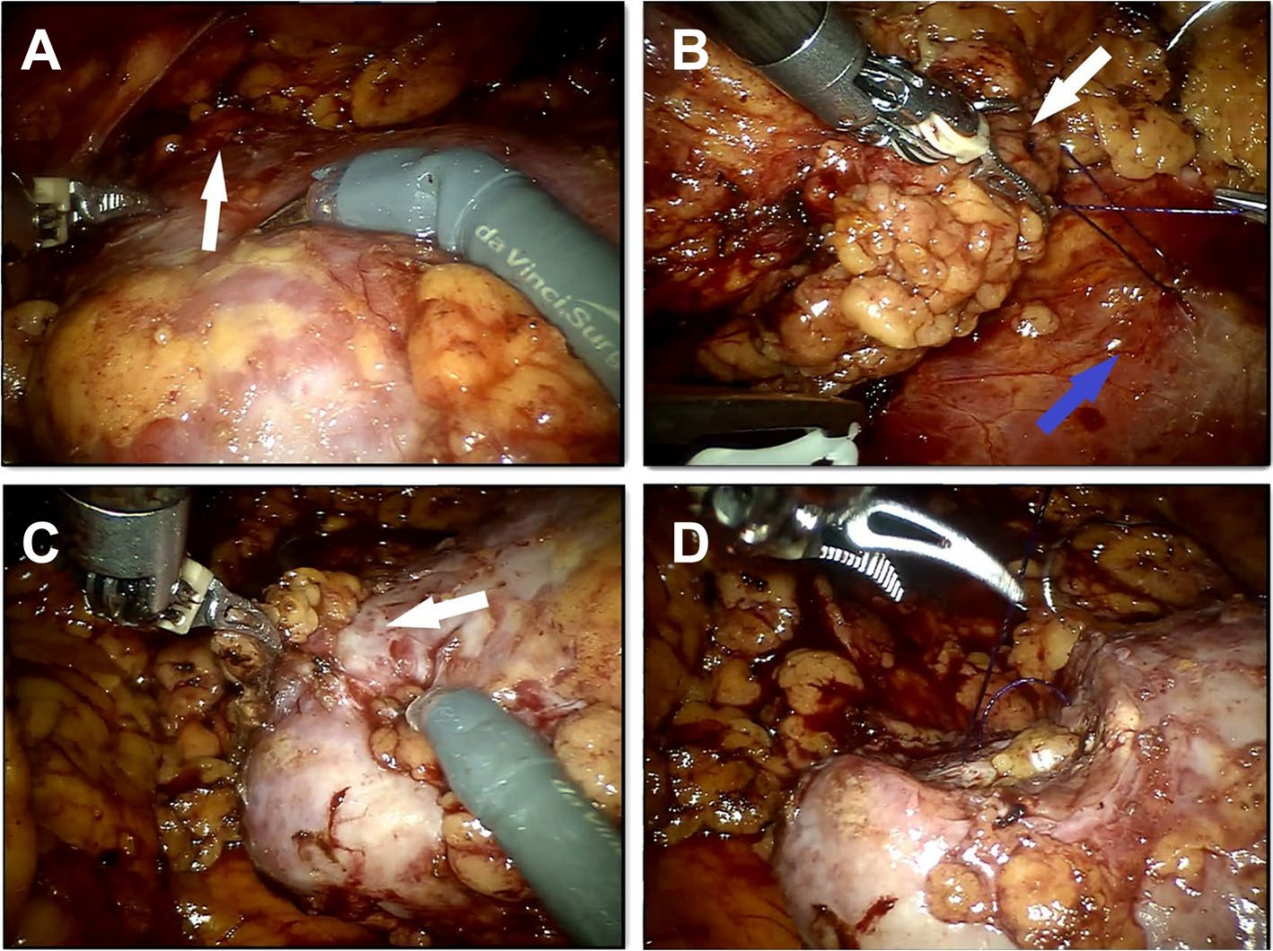
Schematic diagram of the novel technique in rRAPN for renal ventral tumors. **a** Poor tumor (white arrow) exposure. **b** The suture was used to pull the perirenal fat (white arrow) to the psoas major (blue arrow). **c**, **d** The tumor was well exposed during resection and renal reconstruction

All patients underwent da Vinci-assisted partial nephrectomy. The patient was placed in the full flank position and the table fully flexed to increase the space between the 12th rib and iliac crest. The first trocar was placed at the midpoint of the midaxillary line midway between the costal margin and the iliac crest. The retroperitoneal space was first established by blunt dissection, with further extension by a handmade balloon (inflated at 800–1000 mL), and a 12-mm camera port was placed. The two 8-mm robotic instrument ports were placed in the anterior axillary line and posterior axillary line 7–8 cm from the camera port, respectively. After docking, the psoas muscle was identified as the first landmark and the camera was turned so that the muscle was in a horizontal line. Gerota’s fascia was incised after the extraperitoneal fat was removed. Both renal artery and vein were then dissected to allow for adequate closing pressure during cross clamping with bulldog clamps. The perinephric fat along the surface of the kidney was carefully separated. Most of the perirenal fat layer, especially the dorsal fat of the kidney, was removed to enhance the following traction effect. However, the perinephric fat at the lateral edge of the kidney was reserved for traction. The amount of perirenal fat retained depended on the degree of adhesion between the kidney and fat. The ultrasound probe was used to identify the borders and depth of the mass when necessary. Often, renal ventral tumor (Fig. 1) is not satisfactorily exposed (Fig. 2a). The renal artery was clamped using laparoscopic bulldog clamps and then mark the time for WIT.

The perirenal fat was pulled towards the psoas major muscle with a unidirectional barbed suture to make an appropriate surgery field and for an optimal straight tumor exposure (Fig. 2b). A Hem-o-lok clip was applied to maintain the tension of sutures.

Then, the renal tumor and kidney tissue were excised using the scissors with 0.1–0.5 cm from the tumor margin (Fig. 2c). The traction of the perirenal fat on the tumor was maintained during resection. The blood vessels and collecting system were closed with a 2-0 unidirectional barbed suture. After the final tissue bite, a Hem-o-lok clip was applied at the free end of the suture. Then, the second layer suture was continuously performed with a 2-0 unidirectional barbed suture to close the edge of the parenchyma by the same method (Fig. 2d). Additional movie files show the procedure in more detail (see Additional files 1, 2, 3, 4 and 5).

### Statistical analysis

All relevant data were analyzed statistically using Student’s *t*-test, and *P* < 0.05 was considered statistically significant.

## Results

The characteristics and perioperative outcomes of the study cohort are listed in Table 1. There were no significant differences between the two groups in mean age, gender, body mass index, tumor size, operation time, and R.E.N.A.L. score.

**Table 1:** Demographics and perioperative outcomes of patients

Variable	ITT group (*n* = 20)	Traditional technique group (*n* = 8)	*P* value
Age (years)	58.30 ± 12.24	54.75 ± 18.12	0.552
BMI (kg/m^2^)	23.39 ± 4.29	22.01 ± 2.04	0.397
Female (*n*, %)	12 (60.0)	4 (50.0)	0.629
Multiple arteries	8 (40.0)	3 (37.5)	0.717
R.E.N.A.L. score (points)	7.40 ± 1.39	8.00 ± 1.41	0.314
Tumor size (cm)	2.96 ± 1.20	3.79 ± 1.74	0.158
Post-op hospital stays (days)	3.35 ± 0.49	3.25 ± 0.71	0.671
Pre-op Scr (μmol/L)	65.75 ± 15.46	66.00 ± 16.55	0.933
Post-op Scr (μmol/L)	86.40 ± 18.31	87.13 ± 24.82	0.981
Estimated blood loss (mL)	117.45 ± 35.25	324.88 ± 79.42	<0.001
Operation time	154.75 ± 47.42	163.13 ± 69.69	0.715
WIT (min)	17.10 ± 3.14	24.63 ± 4.10	<0.001
Retroperitoneal drainage (mL)	258.75 ± 143.36	314.50 ± 249.23	0.461

*BMI* body mass index, *WIT* warm ischemia time, *Scr* serum creatinine

The mean WIT in the ITT group was 17.10 min which was significantly shorter (*P* < 0.05) than in the traditional technique group at 24.63 min. Estimated blood loss in the traditional technique group was 324.88±79.42 mL, and in the ITT group, it was 117.45±35.25 mL (*P* < 0.05). Suture time and separating kidney time were 1.93±0.29 min and 18.63±3.78 min, respectively. The total duration of the ITT (suture time + separating kidney time) in the ITT group was 20.55±3.72 min.

All the postoperative Scr levels were within normal limits. There were no significant differences between the ITT group and the traditional technique group in postoperative hospital stay and the retroperitoneal drainage (*P* > 0.05).

Pathological characteristics and postoperative complications are summarized in Table 2. On pathology, the rate of clear cell renal cell carcinoma was 90% (*n* = 18) in the ITT group and 87.5% (*n* = 7) in the traditional technique group. No positive surgical margin was found in all cases. All the patients were followed as the recommended schedule. No local recurrence was recorded. One patient in the ITT group underwent urinary tract infection and recovered after 1 week of intervention. No ileus, hemorrhage, perirenal fluid collection, or urine leak occurred.

**Table 2:** Pathological characteristics

	ITT group, *n* (%)	Traditional technique group, *n* (%)
Clear cell RCC	18 (90.0)	7 (87.5)
Papillary RCC	0 (0.0)	1 (12.5)
Chromophobe RCC	2 (10.0)	0 (0.0)
Positive surgical margin	0 (0.0)	0 (0.0)
Urinary tract infection	1 (5.0)	0 (0.0)

*RCC* renal cell carcinoma

## Discussion

Laparoscopic partial nephrectomy has been shown to be an accepted, safe, and feasible treatment option for small localized renal masses [16–18]. With the introduction of the robotic technique, robot-assisted partial nephrectomy has become increasingly widespread for the management of small renal masses. Robot-assisted partial nephrectomy has achieved a decrease in postoperative complications and operative time compared to open partial nephrectomy [19, 20]. The robotic technique allows surgeons to overcome many of the technical challenges of pure laparoscopic surgery thereby shortening the learning curve [21–23]. Robot-assisted partial nephrectomy is demonstrated to be superior to conventional laparoscopic partial nephrectomy in terms of estimated blood loss and WIT, because of the 3D vision and precise dissection of the robotic system [5, 24]. A retroperitoneal approach is more suitable for patients with prior intra-abdominal surgery or multiple arteries [8, 9]. The approach avoided excessive interference of abdominal organs and reduced operation time [6, 25–27]. However, the renal ventral tumor with poor tumor exposure would hinder the tumor resection and extend the WIT, which limits the range of the application of rRAPN.

Feliciano et al. used an additional mechanical arm during rRAPN, which could reduce the complications and positive surgical margins caused by poor exposure [28]. However, this approach consumed additional instruments or assistants, which increased medical cost and reduced the operation space. In order to optimize tumor exposure in rRAPN, we developed a novel internal traction technique.

In this study, the psoas major exerted traction on the kidney during tumor resection and the tumor exposure was improved. With the ITT, we could stabilize the tumor in position and maintain the traction during tumor incision without adding an additional trocar. The study indicates that the WIT was significantly reduced using the new technique. WIT has been considered a significant determinant in postoperative Scr. A WIT of <25–30 min is the widely recommended standard at which any acute kidney injury is considered reversible, and multiple studies have shown worsening functional outcomes associated with WITs >25 min [29–31]. While ITT spent an average of 2 additional minutes for suturing, total WIT decreased. In the present study, mean WIT was 17.10 min in the ITT group, which was considered sufficiently short. Most importantly, shorter WIT may result in better renal function recovery. Separating the kidney without resecting the outer renal edge fatty tissue is the key procedure of the technique. The brittle of adherent perinephric adipose tissues determined the amount of perirenal fat retained [32]. With the approach, the renal ventral tumor was fully exposed without adding an additional trocar and the medical cost was reduced.

With the application of our technique, the precision and stability of the tumor excision were improved, which may reduce the risk of cutting into the tumor capsule. In our study, all the patients who underwent our new procedure had negative surgical margins on histology. There was no difference in complication rates between the two groups. None of the patients showed evidence of local recurrence or metastatic disease at a median follow-up of 10 months.

The limitations of our study include the small sample size and single institution nature. A larger sample size with longer follow-up periods is warranted to confirm the value of the technique.

## Conclusion

Our initial experience suggests that the internal traction method is a safe and feasible procedure for the renal ventral tumors with prior intra-abdominal surgery or multiple renal arteries.

## Supplementary Information

**Additional file 1.** Poor tumor exposure.

**Additional file 2.** Part of perirenal fat layer was removed.

**Additional file 3.** The renal artery was dissected and clamped.

**Additional file 4.** The kidney was pulled to the psoas major.

**Additional file 5.** The tumor was exposed clearly and excised.

## Data Availability

The data that support the findings of this study are available from the corresponding author upon reasonable request.

## Abbreviations

rRAPN: Retroperitoneal robot-assisted partial nephrectomy
ITT: Internal traction technique
WIT: Warm ischemic time
BMI: Body mass index
RCC: Renal cell carcinoma
Scr: Serum creatinine
